# Silica nanoparticles protect rice against biotic and abiotic stresses

**DOI:** 10.1186/s12951-022-01420-x

**Published:** 2022-04-22

**Authors:** Jianfeng Du, Baoyou Liu, Tianfeng Zhao, Xinning Xu, Han Lin, Yatai Ji, Yue Li, Zhiwei Li, Chongchong Lu, Pengan Li, Haipeng Zhao, Yang Li, Ziyi Yin, Xinhua Ding

**Affiliations:** 1 State Key Laboratory of Crop Biology, Shandong Provincial Key Laboratory for Biology of Vegetable Diseases and Insect Pests, College of Plant Protection, Shandong Agricultural University, Taian, 271018 Shandong People’s Republic of China; 2 Yantai Academy of Agricultural Sciences, Yantai, China; 3 College of Life Sciences, Yantai University, Yantai, China

**Keywords:** Silica nanoparticles (SiO_2_ NPs), Rice resistance, *Magnaporthe oryzae*, Salicylic acid, Drought

## Abstract

**Background:**

By 2050, the world population will increase to 10 billion which urged global demand for food production to double. Plant disease and land drought will make the situation more dire, and safer and environment-friendly materials are thus considered as a new countermeasure. The rice blast fungus, *Magnaporthe oryzae*, causes one of the most destructive diseases of cultivated rice worldwide that seriously threatens rice production. Unfortunately, traditional breeding nor chemical approaches along control it well. Nowadays, nanotechnology stands as a new weapon against these mounting challenges and silica nanoparticles (SiO_2_ NPs) have been considered as potential new safer agrochemicals recently but the systematically studies remain limited, especially in rice.

**Results:**

Salicylic acid (SA) is a key plant hormone essential for establishing plant resistance to several pathogens and its further affected a special form of induced resistance, the systemic acquired resistance (SAR), which considered as an important aspect of plant innate immunity from the locally induced disease resistance to the whole plant. Here we showed that SiO_2_ NPs could stimulate plant immunity to protect rice against *M. oryzae* through foliar treatment that significantly decreased disease severity by nearly 70% within an appropriate concentration range. Excessive concentration of foliar treatment led to disordered intake and abnormal SA responsive genes expressions which weaken the plant resistance and even aggravated the disease. Importantly, this SA-dependent fungal resistance could achieve better results with root treatment through a SAR manner with no phytotoxicity since the orderly and moderate absorption. What’s more, root treatment with SiO_2_ NPs could also promote root development which was better to deal with drought.

**Conclusions:**

Taken together, our findings not only revealed SiO_2_ NPs as a potential effective and safe strategy to protect rice against biotic and abiotic stresses, but also identify root treatment for the appropriate application method since it seems not causing negative effects and even have promotion on root development.

**Graphical Abstract:**

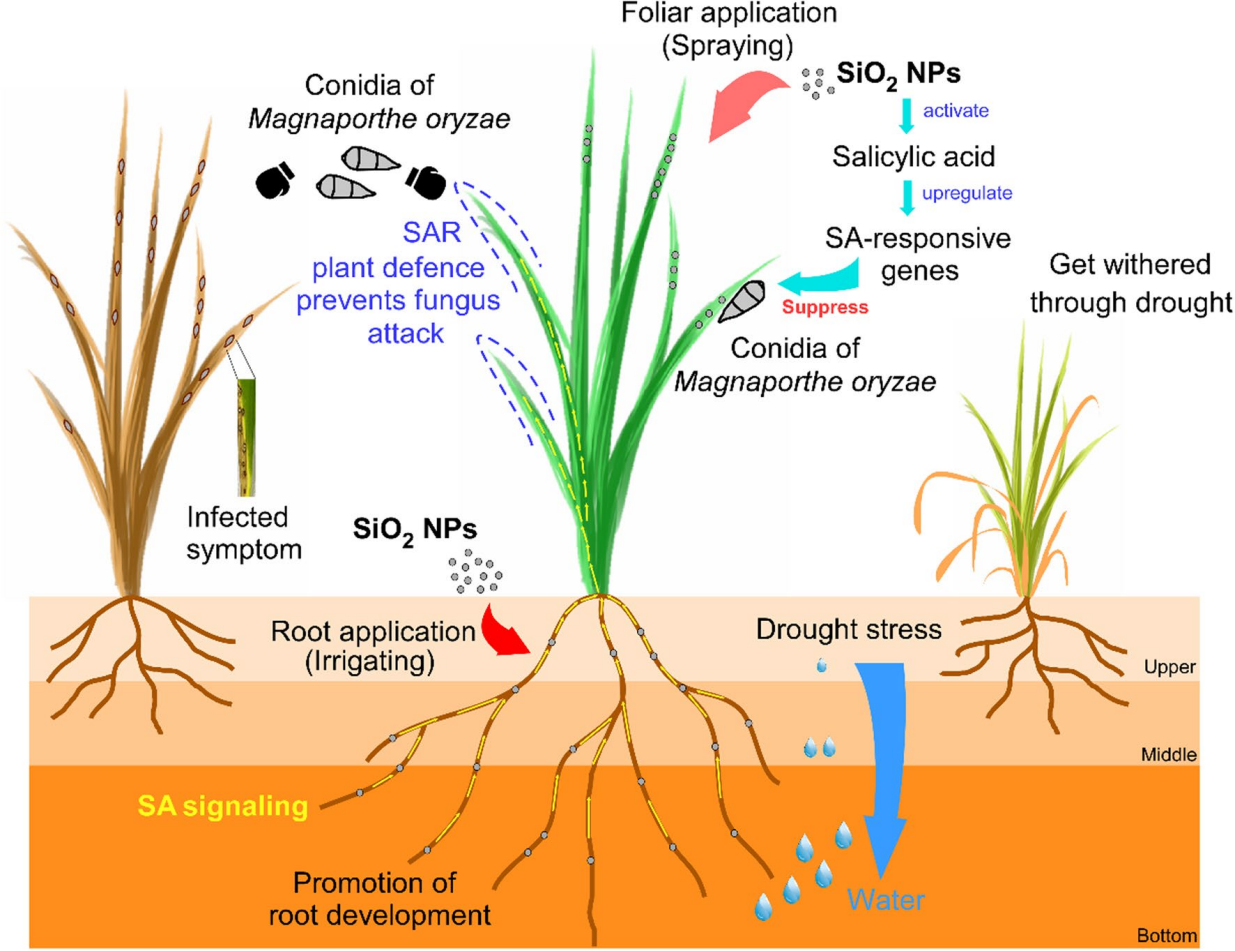

**Supplementary Information:**

The online version contains supplementary material available at 10.1186/s12951-022-01420-x.

## Introduction

Nowadays, despite of people’s diet is getting richer, rice (*Oryza sativa*) is also by far the most essential staple food for more than half of the human population, providing approximately 19% of the daily calories consumed worldwide [1–3]. Studies have shown that by 2050, the world population will increase around 7 to 10 billion, causing sharp increase in rice demand under the guarantee of the growth in global rice yields by 25% before 2030 [2]. Along with these growing demands, rice crops will face several future challenges that will seriously jeopardize its annual production including fungal diseases [4, 5]. Rice blast, caused by the hemibiotroph filamentous fungus *Magnaporthe oryzae*, is one of the most significant threats to the worldwide rice production which contributes to a loss of enough rice to feed 60 million people annually [2, 6, 7]. Unfortunately, neither traditional breeding nor chemical approaches along have been able to contain this disease well and even may cause risk/toxic to human and the environment or fungal resistance with the excessive use of traditional fungicides [6, 8]. Thus, it seems urgent to search for more efficient and safer agent to contribute to the disease control and solve this major threat to global food security.

Nanoagrochemicals, recently, are beginning to attract significant attention as a promising tool to improve yield and global food security, since it has many advantages over conventional products and approaches, which are closely related to enhanced efficacy, reduced input, and lower eco-toxicity [9–14]. Different kinds of nanomaterials have been found to have different uses: (i) working as antimicrobial agents that can directly restrain the virulence of pathogens: nanoparticles of metals such as silver (Ag NPs), copper (Cu/Cu_2_O NPs) and zinc (ZnO/nanocopper composite) had great antibacterial or antifungal capability against *Xanthomonas perforans*, *Fusarium oxysporum*, and *Phytophthora infestans* [15–18]; (ii) functioning as elicitors that can stimulate plant innate immunity to enhance its resistance to the biotic stresses: Ag NPs, Ag-silica hybrid complex and other metal NPs have been found to induce plant immunity by increasing in production of phenolic compounds and oxidative enzymes, as well as up-regulation of systemic acquired resistance marker genes [17, 19, 20]; and also (iii) being made as carriers for active ingredients but not work directly: function as a delivery system of pesticide, micronutrients, and elicitors [13, 21, 22]. Studies have shown that nanomaterials (including Fe_2_O_3_ NPs, TiO_2_ NPs, and carbon-based NPs) at an appropriate dose exhibited the potentiality to suppress pathogen infection and improve plant growth using a model of tobacco (*Nicotiana benthamiana*) and Turnip mosaic virus (TuMV) [23], so that it seems of great importance to identify the effective nanomaterials as well as exploring the specific application concentration. Hence, since the great promise for the use of nanoagrochemicals in plant disease management, it is extremely meaningful to test the disease suppressive effects and effective and safe application methods for different nanomaterials on main food crops like rice.

Silica nanoparticles (SiO_2_ NPs) have been proposed for the controlled nanodelivery of silicon and other active ingredients to plants, however, systematically test about it remains limited. Furthermore, it has been found that silicon is beneficial to plant growth and helps plants overcome biological and abiotic stresses [24, 25]. Rice is a silicon loving crop since it can absorb and accumulate large amount of silicon to the higher level (up to 10%) of shoot dry weight, which is even several times higher compared to those essential macronutrients such as nitrogen, potassium and phosphate [26]. Studies have found that traditional silicon can prevent the infection of *M. oryzae* through not only creating physical barrier to fungal penetration in the leaves and stem owing to the precipitation of biogenic opaline silicon in epidermis, but also potentiating host molecular-scale defenses [27–29]. But even so, it has certain limitations that high concentrations of silicon have the risk of leading chlorosis of leaves, showing kind of phytotoxicity [30]. Thus, studies on whether SiO_2_ NPs could have a positive role on both rice growth and resistance to pathogens without significant toxicity seems quite necessary but are still unknown.

Plants have evolved mechanisms to resist disease that share similar mechanistic principles with the innate immunity of animals to fend off pathogens [31]. A special form of induced resistance is the systemic acquired resistance (SAR) which is characterized by the spread of locally induced disease resistance to the whole plant [32]. SAR can be activated by not only pathogen attack but also the application of elicitor to plant, which can induce signal transduction pathways to promote the signals moving to distant tissues [33, 34]. A pivotal compound contributes to SAR is the plant hormone salicylic acid (SA) which is responsible for the activation of pathogenesis-related (PR) genes [32, 35]. Other factors are synchronously induced during SAR include nitric oxide, reactive oxygen species [36]. Based on the fact that SAR can be stimulated by resistance-inducing compounds instead of direct irreversible genetic modifications or fungicides with potential environmental risks in essential crops such as rice, maize, as well as barely [37, 38], it could be an alternative strategy for controlling crop disease on these crops including rice bacterial leaf blight (*Xanthomonas oryzae* pv. *oryzae*), rice blast (caused by *M. oryzae*), and Fusarium stalk rot (caused by *Fusarium graminearum*) [37, 39, 40].

A recent study reported the potential of SiO_2_ NPs in inducing local and systemic disease resistance in *Arabidopsis thaliana* against the bacterial pathogen *Pseudomonas syringae* [30]. However, the specific mechanistic understanding of the underlying processes and how it works on main crops is still lacking. In this study, we found that SiO_2_ NPs could stimulate plant immunity in order to enhance rice resistance against the rice blast fungus through foliar treatment within an appropriate concentration range. What’s more, this SA-dependent fungal resistance could also be acquired with root treatment through a SAR manner and had a more plant-friendly representation with no phytotoxicity. Interestingly, root treatment with SiO_2_ NPs also promoted the root development of rice seedlings leading to its increased ability of water absorption and a better response to drought. Firstly, our findings identified SiO_2_ NPs as a potential effective and safe strategy to protect rice against biological and abiotic stress in the background of sharply increased grain demand and climate changes. Then, we preliminarily revealed the mechanisms of which different treatment methods led to different effects on rice resistance. Moreover, our results identified root treatment for the appropriate plant-friendly application method since it seems not causing negative effects and even have promotion on root development.

## Materials and methods

### Plant growth conditions

The susceptible rice *Oryza sativa* cv. CO39 and Nipponbare to *M. oryzae* were grown in black soil mixed with vermiculite and Pindstrup substrate. The salicylic acid (SA) defective mutant that overexpressing salicylate hydroxylase (NahG) in Nipponbare background (NIP-NahG) [41] was also used. The rice seeds were washed for more than 5 times with deionized water and germinated on the filter paper in wet dish under 37 °C for 3 days and then sown into the soil. The plants were growth in a 12 h photoperiod with 70% relative humidity with the temperature of 28 °C. Two-week-old rice seedlings were used for the fungus inoculation experiments.

### Culture of *M. oryzae* and conidia production

The *M. oryzae* Guy11 was used as the wild type strain in our study. All *M. oryzae* strains were cultured for vegetative growth on complete medium (CM) within 2 weeks in completely darkness at 28 °C [42–44]. For fungus asexual reproduction (conidia production), the *M. oryzae* strains cut from CM plate was cultured on straw decoction and corn (SDC) agar media at 28 °C for 7 days in darkness and then followed by 3 days of continuous illumination under fluorescent light [45].

### Virulence test

Conidia of Guy11 were harvested from more than 3 plates of 10-day-old SDC agar cultures were filtered through double layers of Miracloth (EMD Millipore Corp., 475855-1R) and resuspended to a concentration of 5 × 10^4^ spores/mL in water solution with 0.2% (w:v) gelatin (Solarbio, G8061). Specific spray inoculation assays referred to our previous studies [46–48].

Multiple evaluation methods were used to systematically judge the rice blast severity, including lesion area, types, as well as relative fungal growth in diseased leaves. For lesion type test, the lesions were divided into 1–5 types according to their severity: type 0, leaves with no lesion at all; type 1, leaves contained pinhead-sized dark specks without obvious centers; type 2, diseased leaves with small brown lesions within 1 mm; type 3, 2 to 3 mm gray spots with brown margins; type 4, elliptical gray spots over 4 mm; type 5, large eyespot lesions that coalesced infecting 50% or more of the leaf area. For the ‘relative fungal growth (RFG)’ test, total DNA was extracted from 1.5 g disease leaves and tested by qRT-PCR (ChamQ™ SYBR® qPCR Master Mix [Vazyme Biotech Company, Q311-02/03]) with *M. oryzae* 28S ribosomal gene (rDNA) and *RUBQ1* primers [43, 49]. The fungal growth inhibition rate was calculated by the percentage of the difference between the relative fungal growth of no-treated control (CK) and different concentration treatments of SiO_2_ NPs to the CK itself [(RFG_CK_-RFG_treatment_)/RFG_CK_*100%] [46].

### SiO_2_ NPs preparation and characterization

The SiO_2_ NPs were synthetized through Stöber process using tetraethyl orthosilicate as silicon source, ammonia as catalyzer and ethanol as solvent [50]. Briefly, 3 mL tetraethyl orthosilicate was dissolved in 50 mL absolute ethanol and made ultrasonic concussion for 25 min prepared for solution A. 150 mL absolute ethanol, 4 mL ultrapure water and 12 mL ammonia (25% NH_3_) were mixed together and then ultrasonic concussion for 15 min prepared for solution B. After stirring solution B at the constant temperature of 50℃ for 10 min, solution A was poured in slowly, reacted for a certain time until the solution is turbid and then started to collect the particles. The particles resulting after 4 h of hydrolysis and polycondensation of tetraethyl orthosilicate were next washed by four times of centrifugation (18,000 ×*g* for 10 min) in ultra-pure water and over five times of dialysis through the membrane with a 14 kDa molecular weight cutoff (regenerated cellulose, Carl Roth). The particles were dried in a vacuum oven at 100 ℃ for more than 2 h, and then used for particle characterization. The particles were then attached to metallic stubs with carbon stickers and sputter-coated with gold for around 30 s and observed and take images with scanning electron microscope (SEM) (Hitachi, SU8100). The size of the particle was then calculated through ImageJ (version 1.52n) analysis according to the SEM micrographs.

### Treatment for rice with SiO_2_ NPs

Different concentration of SiO_2_ NPs (10, 100, 500, 1000, 2000, and 3000 mg/L) were dissolved in ultrapure water. For foliar treatment, SiO_2_ NPs were used to pretreat the 2-week-old rice seedlings (cv. CO-39) by spraying onto the rice leaves two hours before inoculation with *M. oryzae* conidia. For root treatment, total amount of 4 L SiO_2_ NPs were used to treat each sample with nearly 2400 g soil, two hours before inoculation with fungal conidia. Equal amount of water treatment was used for control.

#### Transmission electron microscopy (TEM) observation

Targeted fresh leaves and roots tissues were carefully selected. Sharp blades were used to select and divide fresh tissue blocks quickly within 2 mins and make sure that the size of every tissue block was kept for no more than 1 mm^3^. Before sampling, prepared petri dishes filled with fixative (Servicebio, CR2105174) for TEM in advance. The little tissue blocks were transferred into new EP tubes with fresh TEM fixative for further fixation, meanwhile, kept vacuum extraction until the samples sank to the bottom. The samples were fixed for 120 min and then fixed at 4 ℃ for preservation. And then the divided tissues were fixed with 2.5% glutaraldehyde in phosphate buffer (pH 7.0) for more than 5 h following with repeated washing for more than three times and then fixed with 1% OsO_4_ within the phosphate buffer (pH 7.0) for 1 h, and washed for three times again in the phosphate buffer. Then, the specimen was dehydrated by different concentration of ethanol (30%, 50%, 70%, 80%, 90%, 95% and 100%) respectively for about half to one hour during each step, transferred to the absolute acetone for the next 20 min. Then, the specimen was placed in 1:1 mixture of absolute acetone and the final Spurr resin mixture for one hour at 26 ℃; then transferred to 1:3 mixture of the above mixture for three hours and to final Spurr resin mixture for overnight. Specimen was placed in capsules contained embedding medium and heated at 60 °C for 48 h. The specimen sections were stained by uranyl acetate and alkaline lead citrate for 20 min respectively and detected under TEM (Hitachi, HT7800).

#### Determination of SA level

Fresh materials were frozen in liquid nitrogen and lyophilized. Sample processing and preparation according to [51]. Data analysis was performed using UPLC-ESI–MS/MS system (UPLC, ExionLC AD; MS, Applied Biosystems 6500 Triple Quadrupole). The chromatography system was connected to AB 6500 + QTRAP LC–MS/MS System, equipped with an ESI Turbo Ion-Spray interface. For process quantitative data and plant samples from calibration standards, the MASSLYNX NT software version 4.1 (Micromass) was taken into use.

#### Total silicon content analysis

The collected samples were rinsed with deionized water, dried in an oven at 105 °C for about 20 min, and then dried at 75 °C to the constant weight. Crush the dried sample, then weigh 0.1000 g of the dried sample that has passed through a 60-mesh sieve into a 50 ml polypropylene plastic tube, add 5 ml of 40% sodium hydroxide and 5 ml of water, and mix well. The above samples were placed in a sterilization pot at 121 °C for 20 min. Then add 5 mL of 5 M sulfuric acid to the sample and add water to 40 mL. The processed samples were measured for silicon content by molybdenum blue colorimetry [52].

#### Extraction of plant DNA

The plant leaves (twenty leaves for each treatment) after inoculating fungus were quickly frozen in liquid nitrogen and were homogenized with the ceramic mortar and pestle. Total DNA was then extracted through conventional CTAB (hexadecyltrimethylammonium bromide) assays. The homogenized leaf samples were added with 500 μL CTAB extraction buffer (2% CTAB [w/v, Sigma-Aldrich, H5882], 1 M Tis-HCl [Sigma-Aldrich, PHG0002], 0.5 M EDTA [Sigma-Aldrich, E9984], NaCl [Sigma-Aldrich, S9888], pH 8.0) and incubated at 65 °C water bath for one hour and gently mixed every 20 min. After heating for one hour, added 0.8 mL of chloroform/isoamylalcohol (24:1) solution and gently mixed the tube. Then centrifugated for 15 min (14,000 ×*g* at 4 °C) and carefully transfer the aqueous phase to a new EP tube. Added 1 µL RNase (DNase-free) and incubated for 30 min at 37 °C. Added double volume of ethanol, and gently inverted the tube to mix completely. Left to precipitate for more than 4 h at − 20 °C and then centrifugated for 15 min (14,000 × g at 4 °C). Finally, DNA was washed by 70% ethanol and dried in the fume hood, then resuspended the total DNA with sterile water.

#### Plant RNA extraction and quantitative RT-PCR analysis

Plant leaves or roots of twenty rice seedlings were used to be frozen in liquid nitrogen and were homogenized with the ceramic mortar and pestle. Then, homogenized powder of 0.1 g for each sample (both leave and root) was used for further total RNA extraction using a plant total RNA extraction kit (Sigma Life Science, STRN50). For qRT-PCR, total RNA was reverse transcribed into first-strand cDNA using the HiScript II Q RT SuperMix for qPCR (Vazyme Biotech Company, R233-01). The qRT-PCR was run on the Applied Biosystems 7500 Real Time PCR System with ChamQ SYBR® qPCR Master Mix (Vazyme Biotech Company, Q311-02/03). Normalization and comparison of mean Ct (Cycle threshold) values were performed as previously described [49].

### Drought-tolerance assays and water loss rate measurement

For testing the drought stress tolerance in soil, 2-week-old rice seedlings with normal water supply were used. 3000 mg/L concentration of SiO_2_ NPs (4 L totally) were used to treat with the rice seedlings and equal amount of water was used in CK group as control. After the treatment, the rice seedlings began to be drought with no water supply for the next 20 days and then photographed. For water loss rates, the leaves of rice seedlings after drought treatment were detached and placed at room temperature. The fresh weight of the detached leaves was monitored at the indicated time points. Water loss was calculated from the decrease in the fresh weight compared with time zero. The average water loss rate was calculated from three independent experiments [53].

## Results

### Particle size distribution of silica nanoparticles and its toxicity to *M. oryzae*

**Fig. 1:**
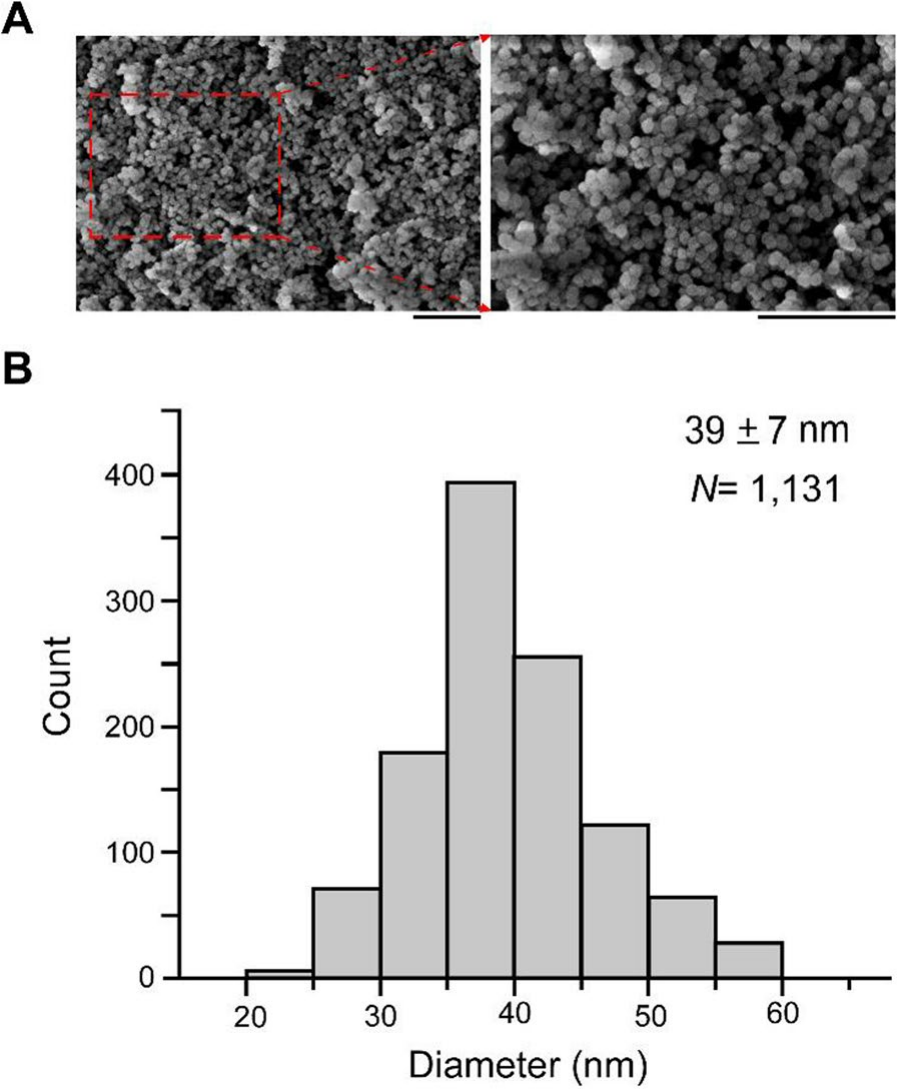
SiO_2_ NPs under investigation. **A** Scanning electron microscope (SEM) images of the particle. Scale bar, 500 nm. **B** Particle size distribution based on the SEM image analysis. Averages ± standard deviations

Whether silica nanoparticles (SiO_2_ NPs) are effective on controlling rice blast and its specific mechanism have not been reported yet. Herein, the SiO_2_ NPs were synthetized through Stöber process [50] using tetraethyl orthosilicate as silicon source, ammonia as catalyzer and ethanol as solvent, then observed by scanning electron microscopy (SEM). SEM observation confirmed that the SiO_2_ NPs we synthetized had the primary particle size was 39 ± 7 nm (average ± standard deviation) (Fig. 1A, B).

To investigate if SiO_2_ NPs have a role in plant resistance to fungus, we used rice, the most important staple food around the world, and rice blast fungus, which caused significant threat to rice production for further study. What’s more, the rice- rice blast fungus model has become one of the essential patterns studying the interaction between plant and microorganism these days. We firstly tested whether SiO_2_ NPs have direct toxic effect on fungi growth. The wild-type rice blast fungus *Magnaporthe oryzae* (Guy11) was cultured on complete medium (CM) with or without SiO_2_ NPs at 10, 100, 1000, 3000 mg/L, respectively. Meanwhile, the formation rate of appressorium, a special structure that employs enormous turgor pressure to rupture rice leaves for infection, was also measured. At these concentrations, neither the growth nor the conidia germination and appressorium formation rate were harmed or limited (Additional file 1: Table S1).

### Exogenous foliar treatment of SiO_2_ NPs confers rice resistance to the rice blast fungus *M. oryzae*

**Fig. 2:**
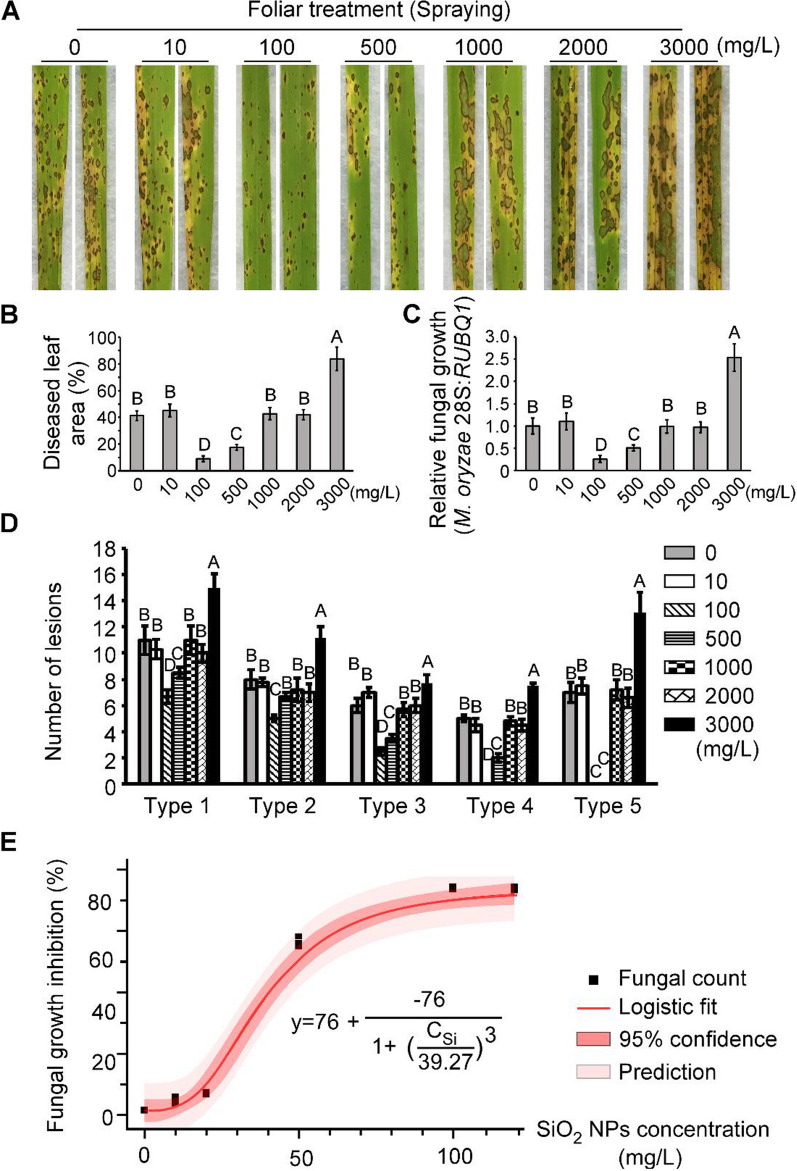
SiO_2_ NPs enhance rice resistance to *M. oryzae* through foliar treatment. **A** Rice spraying assays. Rice leaves were pretreated with different concentrations of SiO_2_ NPs two hours before spraying *M. oryzae* conidial suspension (5 × 10^4^ spores/mL) on two-week old rice seedlings. **B** Diseased leaf area analysis. Data are presented as a bar chart showing percentage of lesion areas analyzed by Image J. **C** Severity of blast disease was evaluated by quantifying *M. oryzae* genomic 28S rDNA relative to rice genomic Rubq1 DNA (7 days post-inoculation). Mean values of three determinations with standard deviations are shown. **D** Quantification of lesion types (per 1.5 cm^2^) on susceptible rice spayed with conidia of wild type *M. oryzae* strain*.* Disease lesions were quantified by a ‘lesion-type’ scoring assay which divided the lesions into 1–5 types according to their severity. Error bars represent SD and different capital letters represent significant differences (P < 0.01). **E** SiO_2_ NPs triggered dose-dependent fungal inhibition in a concentration range 7 days after inoculation of wild type *M. oryzae* strain on susceptible rice cv. CO-39. Fungal growth inhibition rates data from Additional file 1: Fig. S1 were used to establish a logistic dose–response model. Above the dynamic range, the fungal infection could increase again (Fig. 2A–C). C_Si_, SiO_2_ NPs concentration in mg/L

Since SiO_2_ NPs had no obvious toxicity on the fungus, we thus tested if SiO_2_ NPs could enhance the resistance of rice to *M. oryzae* by stimulating plant defense response. Different concentrations of SiO_2_ NPs (10, 100, 500, 1000, 2000, and 3000 mg/L) were used to pretreat the 2-week-old rice seedlings (cv. CO-39) by spraying onto the rice leaves 2 h before inoculation with *M. oryzae* conidia. Fungal conidial suspensions (5 × 10^4^ spores/mL) were then used to spray onto the rice leaves. Results showed that both relatively low (10 mg/L) and higher (1000, 2000 mg/L) concentrations had no significant effect on rice resistance to *M. oryzae*, whereas 100 and 500 mg/L SiO_2_NPs treatment could substantially reduce infectious fungal growth and limit the lesion area (Fig. 2A–C). Furthermore, the lesions were quantified by a ‘lesion-type’ scoring assay which divided the lesions into 1–5 types according to their severity and found that 100 and 500 mg/L SiO_2_ NPs treatment also reduced the lesions in every types even eliminate type 5 lesions (Fig. 2D). Among the several concentrations, 100 mg/L plant treatment showed the best inhibition effect since it reduced the diseased area and relative fungal growth to just only 25% of the no treated control (Fig. 2A–C), meanwhile, 5 mL SiO_2_ NPs were used for one pot (containing 20 rice seedlings) treatment and it seemed that the final dosage was 0.025 mg/plant. These results indicated that exogenous foliar treatment of SiO_2_ NPs could confer rice resistance to the fungus with a quite low concentration.

We further tested more denser gradient around the effective concentration 100 mg/L. Using a standard logistic model, we found a dose-dependent manner between the inhibitions of infectious fungal growth and SiO_2_ NP concentration under the dynamic range of 120 mg/L (Fig. 2E and Additional file 1: Fig. S1). These results indicated that the SiO_2_ NPs induced resistance to *M. oryzae* was functional within a suitable relative low concentration range and SiO_2_ NPs had the potential for effective disease control. We also found that no phytotoxicity on plant leaves under neither low nor higher concentrations of SiO_2_ NPs (Additional file 1: Fig. S2), suggesting foliar treatment of SiO_2_ NPs was relative safe to the plant.

Although the SiO_2_ NPs-induced resistance to *M. oryzae* seems quite effective within a dynamic range (Fig. 2E), we surprisingly found that when the concentration reached to 3000 mg/L, it could lead to increased fungal infection and thus less effective in activating rice defense (Fig. 2A–D), indicating that exogenous foliar treatment of SiO_2_ NPs could confer rice resistance to *M. oryzae* but needs an effective concentration.

### Exogenous root treatment of SiO_2_ NPs also enhances rice resistance with even better effect

**Fig. 3:**
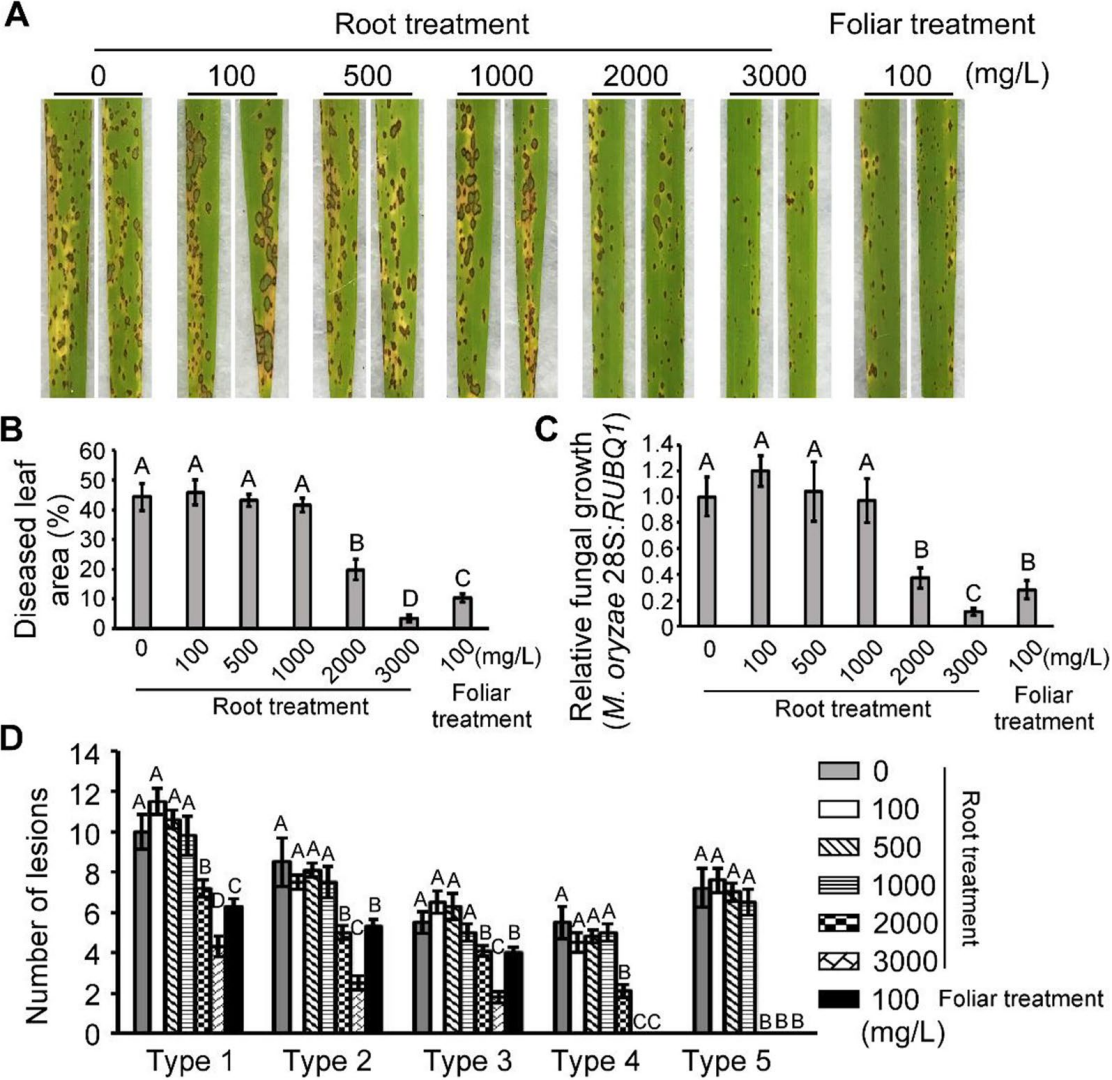
Root treatment with SiO_2_ NPs also enhances rice resistance. **A** Rice spraying assays. Rice seedlings pretreated with different concentrations of SiO_2_ NPs through irrigating method 2 h before fungus inoculation. **B** and **C** Diseased leaf area and disease severity analysis. Lesion areas were analyzed by Image J. and disease severity was evaluated by quantifying *M. oryzae* genomic 28S rDNA relative to rice genomic Rubq1 DNA. **D** Quantification of lesion types (per 1.5 cm^2^) on susceptible rice spayed with conidia of wild type *M. oryzae* strain*.* Error bars represent SD and different capital letters represent significant differences (P < 0.01)

In order to avoid the weakening effect during higher concentrations of foliar treatment, we curious about if changing the application method could improve this limitation. Considering that the uptake of traditional silicon mostly takes place through plant roots as silicic acid [28], we then used irrigating method to make the root treatment with the SiO_2_ NPs concentration of 100, 500, 1000, 2000, 3000 mg/L, respectively. Surprisingly, we found that irrigating of SiO_2_ NPs over the concentration of 2000 mg/L had a significant weakness of the disease severity and had a better effect at 3000 mg/L (reducing the diseased area and relative fungal growth to around 10% than the non-treated control) rather than that 100 mg/L of foliar treatment (Fig. 3A–D), with no negative effects on the resistance, suggesting that exogenous irrigating for root treatment of SiO_2_ NPs seems a more effective and safer measure for preventing the rice blast. Although the high concentration reached to 3000 mg/L for root treatment, actually we used 4 L SiO_2_ NPs for treating nearly 2400 g soil which means that the total application dosage was 5 mg/g, within a reasonable range of use according to other studies [54–58].

### The intake of SiO_2_ NPs in rice leaves and roots

**Fig. 4:**
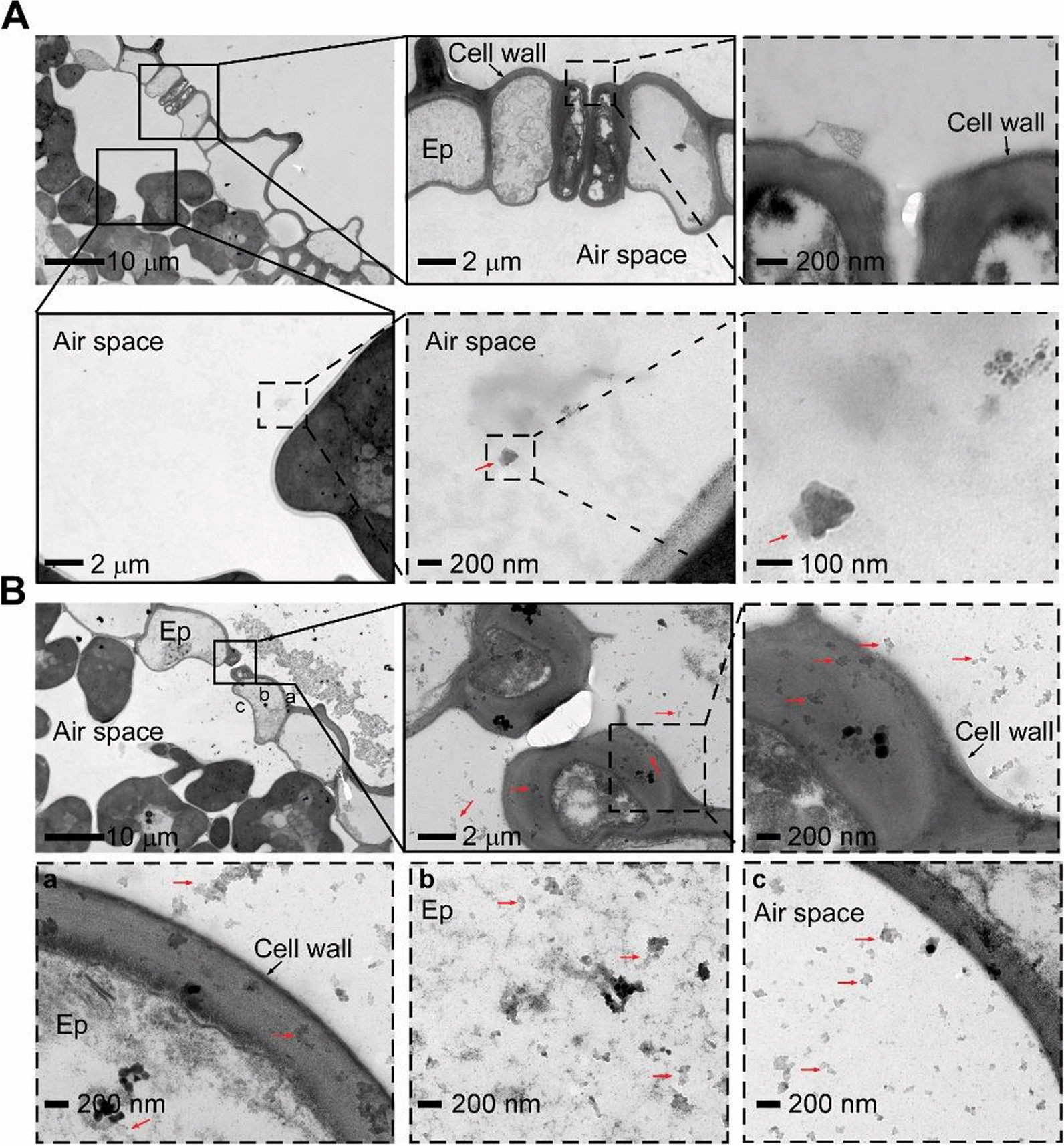
TEM observation of SiO_2_ NPs distribution in rice leaves under different concentrations through foliar treatment. Leaves of the 2-week-old rice seedlings were exposed to SiO_2_ NPs with spraying method treated for 1 day before observation. Red arrows point to the nanoparticles. Ep stands for epidermis. The boxes with dashed and solid lines represent the magnification of the part. **A** Rice leaves treated with 100 mg/L SiO_2_ NPs through foliar treatment. Little SiO_2_ NPs were observed in the inside air space with no nanoparticles accumulated around the stomata. No nanoparticles found in the cell wall and epidermis cells. **B** Rice leaves treated with 3000 mg/L SiO_2_ NPs through foliar treatment. Numerous nanoparticles accumulated around the stomata and entered into the plant not only through the stomata but also through the nearby epidermis cells. The small letter “a, b, and c” point to the SiO_2_ NPs entry process from outside cell wall to the inside epidermis cell and then into the air space

**Fig. 5:**
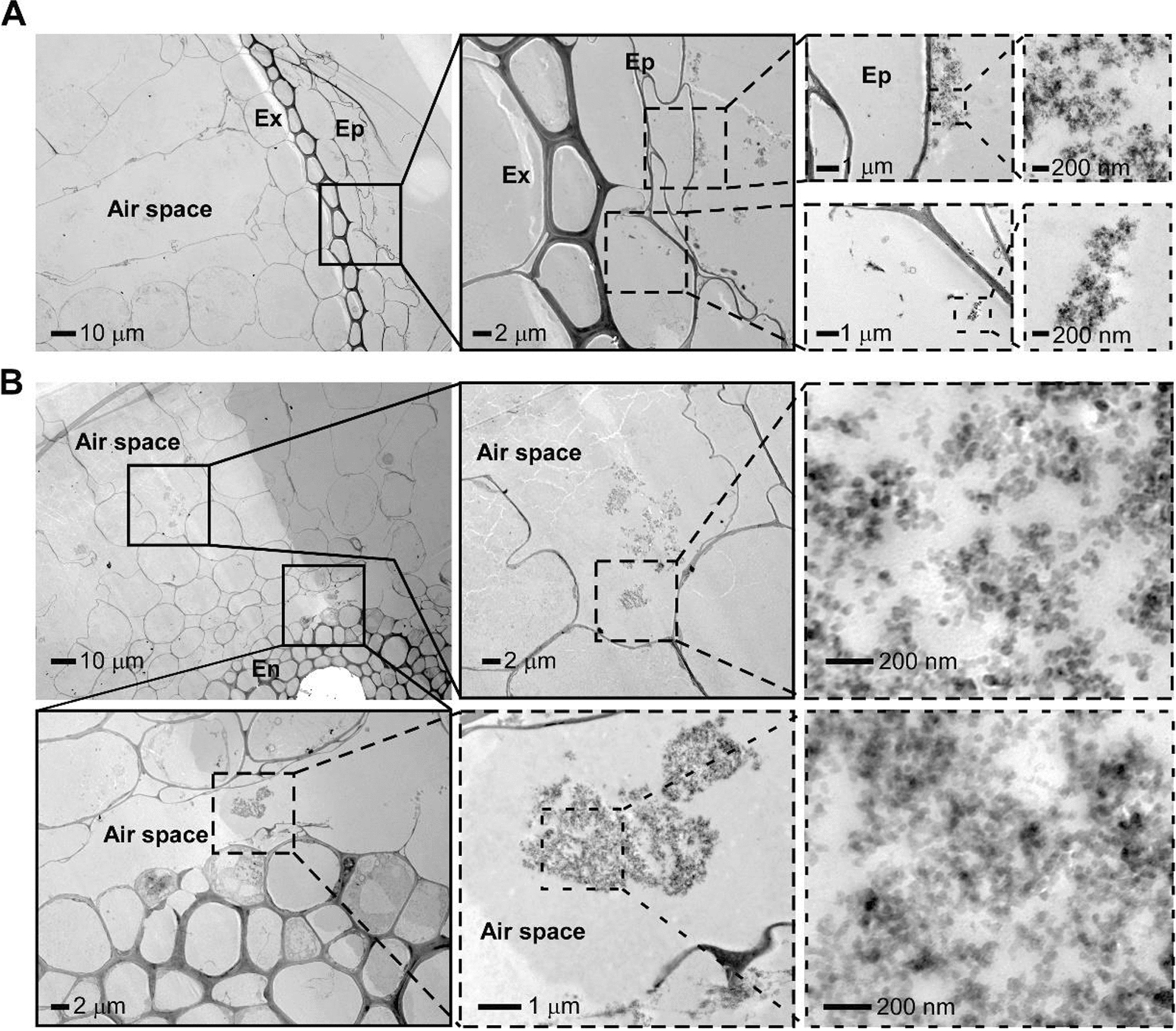
TEM observation of SiO_2_ NPs distribution in rice roots through root treatment. Roots of the 2-week-old rice seedlings were exposed to 3000 mg/L of SiO_2_ NPs treated for 1 day before observation. Ep stands for epidermis, Ex stands for exodermis, En stands for endodermis. The boxes with dashed and solid lines represent the magnification of the part. **A** Outer part of the rice root. Massive nanoparticles accumulated outside the root epidermis and only some of them could be absorbed into the inner side of epidermis. **B** Inner part of the rice root. SiO_2_ NPs could be transported through the root air spaces

The interaction of the nanoparticles with rice was assessed by TEM in both leaves (Fig. 4) and roots (Fig. 5) 1 day after application of SiO_2_ NPs. Results showed that the size around 40 nm of SiO_2_ NPs with 100 mg/L of foliar treatment might allow them to enter the rice leaf through the stomata and distributed within the large extracellular air space without penetrating any cell walls, with no nanoparticles accumulated outside the stomata (Fig. 4A), compared with the non-treated rice leaf cells control (Additional file 1: Fig. S3). This finding was in line with that the SiO_2_ NP intake is clearly restricted to the stomata and the extracellular spongy mesophyll on *A. thaliana* leaves due to the impermeable barrier function of leaf cuticle to nanoparticles [30, 59]. However, we further found that high concentration of SiO_2_ NPs at 3000 mg/l foliar treatment led to massive accumulation around the stomata and might destroy the function of stomatal and around epidermis cells which allowed the nanoparticles enter not only through the stomata but also the nearby cells (Fig. 4B). This kind of disordered intake might result in the increased fungal infection in Fig. 2.

In contrast, high concentration at 3000 mg/L of root treatment did not cause disordered absorption since lots of SiO_2_ NPs could be stopped outside the epidermis and only some of them could be intake into the inner side of epidermis (Fig. 5A). Meanwhile, nanoparticles did not be detected inside cells but found in root air space (Fig. 5B) compared with the non-treated rice root cells control (Additional file 1: Fig. S4), indicating that SiO_2_ NPs could transport through rice root air space after intake and the barrier effect of several tissues (including epidermis, exodermis, endodermis and xylem) might make the intake more moderate which could confer rice resistance without negative effect of excessive absorption. Our results were in line with the findings in some other important crops (wheat, maize and lupin) as well as *Arabidopsis* that the nanoparticles enter the roots via symplastic or apoplastic routes and the entry might take place through the pores in the cell wall of the root cells [60], but this kind of moderate intake in rice roots was the first time.

Also, we tested the content of plant total silicon with different SiO_2_ NPs concentrations in different application methods. Compared with the untreated control, 100 mg/L foliar treatment (1.51-fold) and 3000 mg/L root treatment all led to increased silicon and root treatment got more (1.98-fold), and 3000 mg/L foliar treatment caused the most silicon intake (4.77-fold) (Additional file 1: Fig. S5), suggested that 3000 mg/L foliar treatment did cause too much SiO_2_ NPs intake which might result in increased fungal infection and less effective in activating rice defense whereas 3000 mg/L root treatment reduced the intake of nanoparticles.

### SiO_2_ NPs-induced rice resistance to *M. oryzae* depends on salicylic acid

**Fig. 6:**
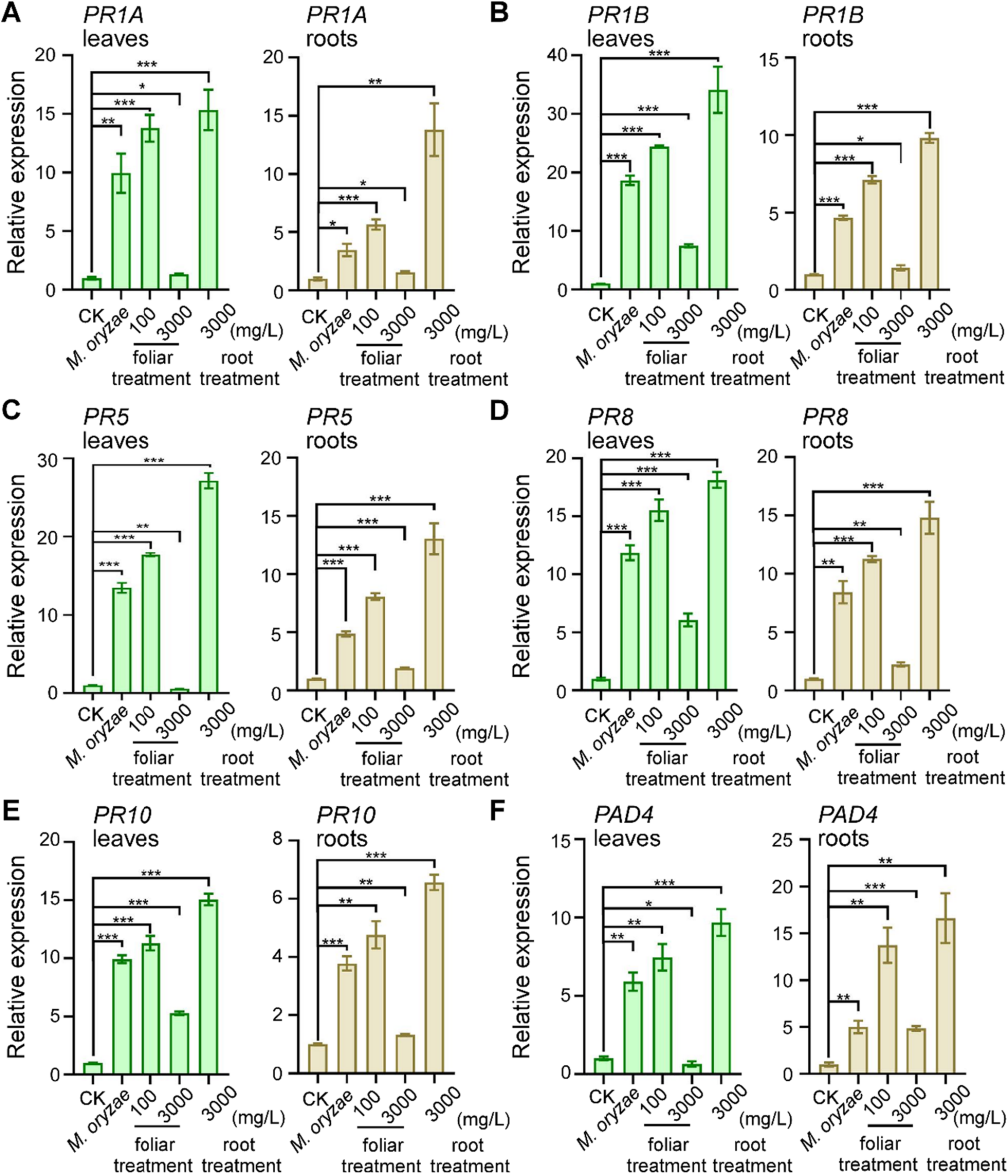
SiO_2_ NPs induce up-regulation of SA-responsive marker genes during infection of *M. oryzae.* RT-qPCR analysis of the gene expression of the SA-responsive genes *OsPR1A* **A** *OsPR1B* **B** *OsPR5* **C** *OsPR8* **D** *OsPR10* **E** and *OsPAD4* **F** in response to different concentrations and treatments of the wild-type rice CO39. Leaves and roots were pretreated 2 h before inoculation with *M. oryzae* conidia. Samples were collected 24 h after fungus infection. Error bars represent SD. *** stands for significant difference P < 0.001, ** stands for significant difference P < 0. 01, * stands for significant difference P < 0.05

Since the finding that irrigating of SiO_2_ NPs to the root of rice could also activate plant resistance of rice leaves to *M. oryzae*, we considered this a kind of induced resistance called systemic acquired resistance (SAR) which is characterized by the spread of locally induced disease resistance to the whole plant [32, 61]. One of the key signaling compounds that contributes to SAR is the plant hormone salicylic acid (SA) and plant SA plays a core regulatory role in plant immunity [32, 35, 62]. We thus quantified the expression of SA-responsive marker genes (*PR1A*, *PR1B*, *PR5*, *PR8*, *PR10*, *PAD4*) under SiO_2_ NPs treatment and *M. oryzae* infection in both leaves and roots, using non-treated rice as control. Results showed that, infection of *M. oryzae* highly induced the expressions of SA-responsive genes compared with the non-treated control (Fig. 6). Meanwhile, SiO_2_ NPs pre-treatment with 100 mg/L foliar treatment and 3000 mg/L root treatment both resulted in higher up-regulation of SA-responsive genes. Moreover, 3000 mg/L concentration of root treatment had better effect in expression of SA-responsive genes in both leaves and roots (Fig. 6A–F), consistent with the results that 3000 mg/L concentration of irrigating SiO_2_ NPs caused better resistance to the fungus. We also found that 3000 mg/L concentration of foliar treatment caused disorder of SA-responsive genes regulations (the up-regulation latitude showed significantly lower than that of fungus inoculation alone) that response to *M. oryzae* inoculation which might result in its increased fungal infection (Fig. 6A–F).

**Fig. 7:**
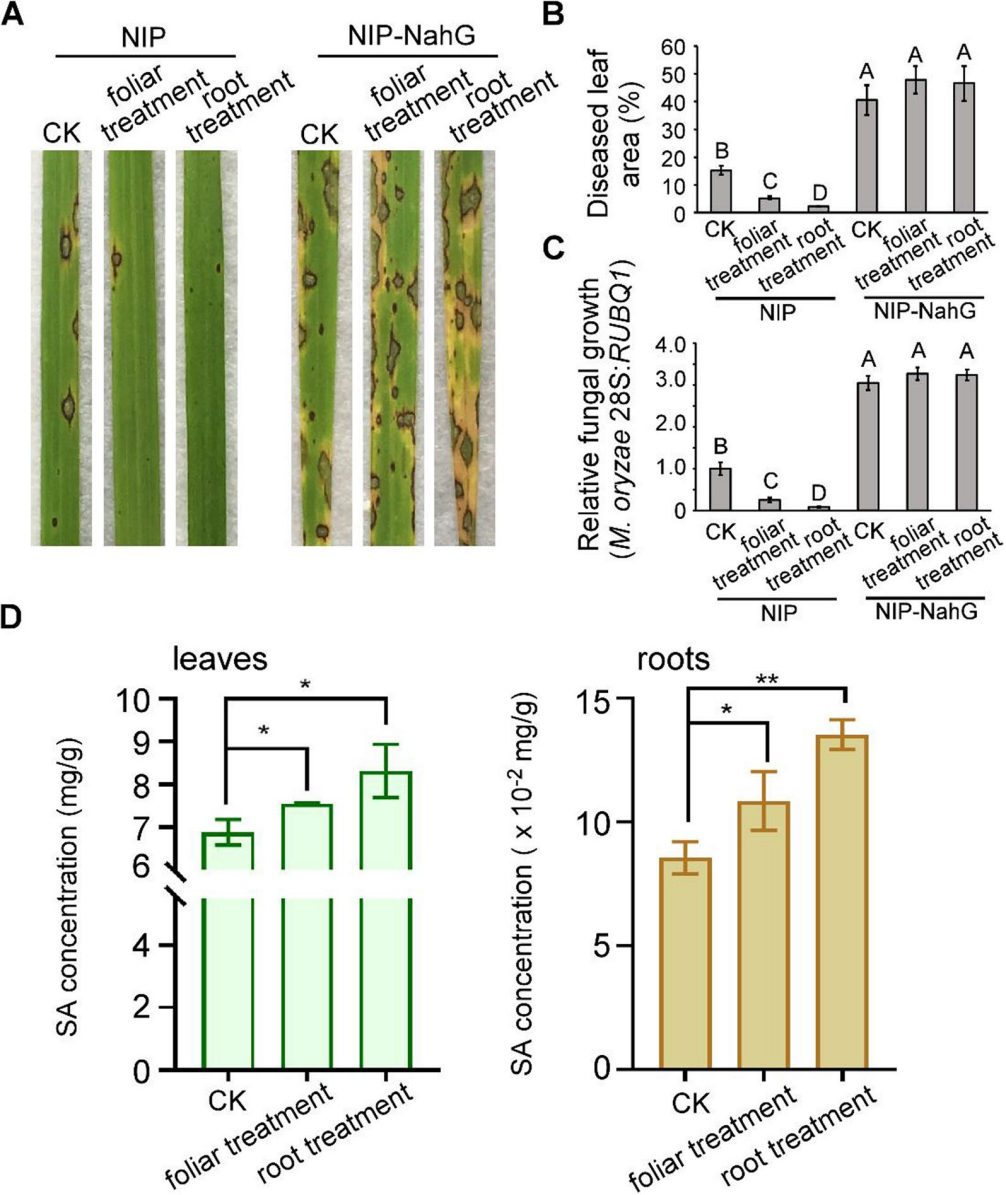
SiO_2_ NPs induced rice resistance to *M. oryzae* based on SA pathway. **A** Rice spraying assays. Rice leaves were pretreated with SiO_2_ NPs two hours before spraying *M. oryzae* conidial suspension (5 × 10^4^ spores/mL) on both two-week old wild-type rice Nipponbare (NIP) and SA defective mutant (NIP-NahG). **B** and **C** Diseased leaf area and disease severity analysis. Lesion areas were analyzed by Image J. and disease severity was evaluated by quantifying *M. oryzae* genomic 28S rDNA relative to rice genomic Rubq1 DNA. **D** The SA concentration in rice leaves and roots were detected with the treatment of 100 mg/L foliar treatment and 3000 mg/L root treatment. Error bars represent SD. ** stands for significant difference P < 0. 01, * stands for significant difference P < 0.05

To further check that SiO_2_ NPs stimulate SAR through SA-dependent pathway or not, we tested the ability of SiO_2_ NPs to induce rice resistance against *M. oryzae* in SA defective mutants that overexpressing salicylate hydroxylase (NahG) in Nipponbare background (NIP-NahG) [41]. Notably, neither SiO_2_ NPs-induced basal resistance to *M. oryzae* (through foliar treatment directly) nor SAR (through irrigating treatment on roots) in NIP-NahG mutant plants were totally blocked, whereas the resistance was normally induced in the wild-type plants (Fig. 7A–C), indicating that SA-dependent defence signaling pathway is essential for SiO_2_ NPs-induced disease resistance. Simultaneously, we detected the SA level in both rice leaves and roots under effective pre-treatments to induce rice resistance (100 mg/L concentration of foliar treatment and 3000 mg/L concentration of root treatment) before inoculating *M. oryzae*, no-treatment with fungus inoculation alone was used as control. Consistent with the results above, 100 mg/L concentration of foliar treatment and 3000 mg/Lconcentration of irrigating SiO_2_ NPs could both increase SA levels in rice leaves as well as roots compared with *M. oryzae* inoculation alone, and 3000 mg/L concentration of root treatment was even higher (Fig. 7D). These results above indicated that SiO_2_ NPs activated SA-dependent defence reactions and exogenous root treatment had a more significant effect.

According to our results, 3000 mg/L concentration of foliar treatment on rice resulted in disordered intake of nanoparticles (Fig. 4) then caused excessive accumulation of silicon in plant (Additional file 1: Fig. S5) which resulted in rough regulation of SA-responsive genes (Fig. 6) and finally increased fungal infection. Different from our findings, in *Arabidopsis*, high concentration of SiO_2_ NPs also attenuates the induced resistance on plant but is still effective on conferring plant immunity, probably due to the excess release of the orthosilicic acid causing oxidative stress or highly intense clogging of the stomata [30]. Other kinds of nanoparticles like elemental sulfur nanoparticles also shows phytotoxicity through excessive use which might owing to the function of antioxidative system imbalance [63, 64].

### Exogenous root treatment of SiO_2_ NPs promotes rice root development and enhance the ability of water absorption to deal with drought

**Fig. 8:**
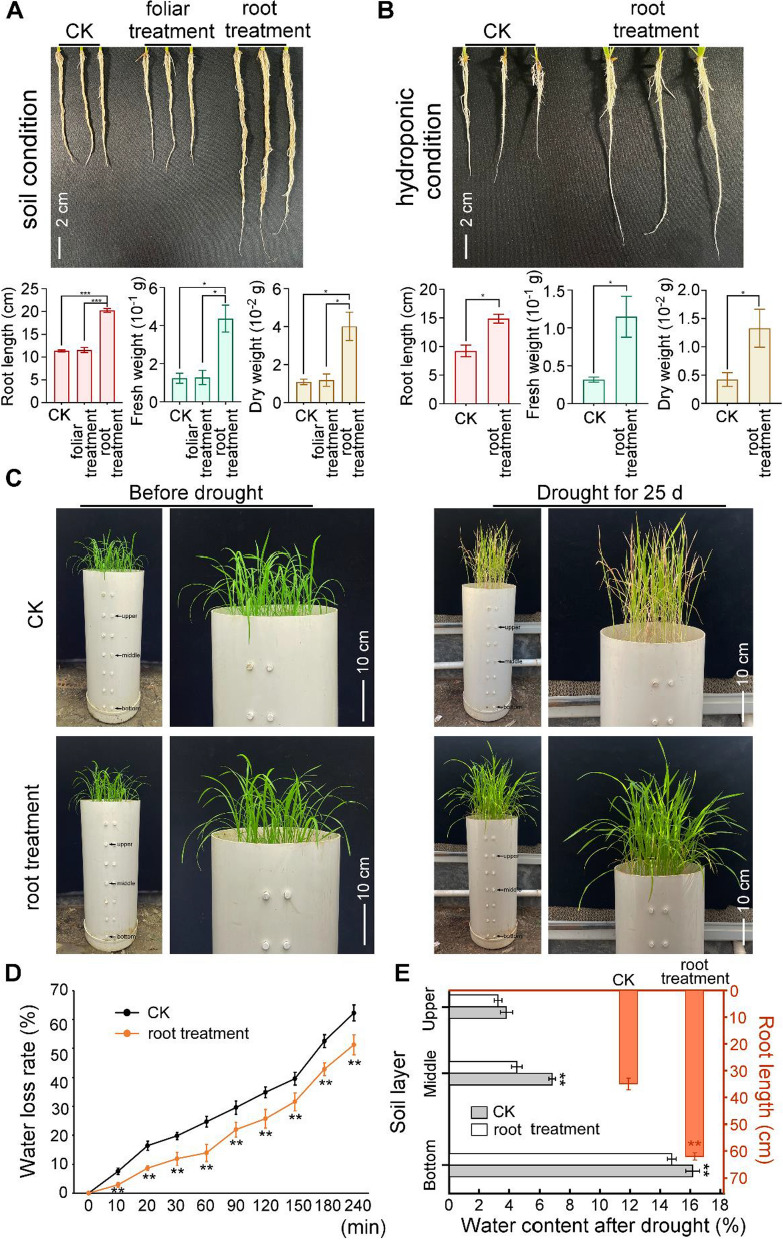
Root treatment with SiO_2_ NPs promote root development of rice and induce resistance to drought. **A** Root development in soil. Rice seedlings cultured in soil condition and treated with SiO_2_ NPs in different application methods. The length, fresh weigh and dry weight of the roots were tested 7 days after treatment. **B** Root development in hydroponic condition. The length, fresh weigh and dry weight of the roots were tested 7 days after treatment. **C** Images showing the phenotypes of the water treated control (CK) and the plant with root treatment of SiO_2_ NPs under drought stress for 20 days. **D** Water loss rates of detached leaves from (**C**). **E** The water content for different soil layers (the ordinate on the left and the abscissa below) and root length (the ordinate on the right and the abscissa above) from (**C**). Error bars represent SD. ** stands for significant difference P < 0. 01, * stands for significant difference P < 0.05

During our collection of root samples under SiO_2_ NPs treatment above, we surprisingly found that the roots with 3000 mg/L concentration root treatment were longer and thicker than the control, whereas other treatments did not show any significant changes. We thus further tested the roots development with 3000 mg/L root treatment (5 mg/g soil) under both hydroponic and soil conditions. Results in different conditions all showed that 3000 mg/L concentration of root treatment promoted the development of rice roots (Fig. 8A, B), consistent with the significant change of root growth related gene expressions (Fig. 8C). Concurrently, we also found that foliar treatment did not promote rice root development, suggested that this kind of promotion of root development could not be activated through signal transmission from leaf to root.

We then curious about if the promotion of roots can enhance the ability of water absorption in order to gain better resistance to drought. Special deep containers with removable little rubber plugs on the side of different depth were used to culture rice seedlings (Additional file 1: Fig. S6). 2-week-old rice seedlings were treated by 3000 mg/L concentration of SiO_2_ NPs through irrigating (treated with water as control) and then began drought treatment for the next 20 days. We detected moisture content at the upper, middle and lower layer of soil through sampling soil from the removable little rubber plug on the side of the containers at the beginning and end of drought treatment (Additional file 1: Fig. S7 and Fig. 8E). Results showed that the rice seedlings treated with 3000 mg/L concentration of SiO_2_ NPs with irrigating to root became withered slowly along with lower leaf water loss rate than the control (Fig. 8C, D). The soil moisture content decreased to less than 8% in both upper and middle soil layers which caused the withered of CK since its roots could only reach the depth of middle soil layers, whereas root treatment with SiO_2_ NPs led to promotion of root development that allowed the root to reach bottom soil layer in order to absorb water in depth (Fig. 8E). Considering that roots play vital function in plant life cycle since they not only provide anchorage for plants growing aboveground, regulate the uptake of water and essential nutrients from the soil, but also act as storage of resources [65], we believe that this kind of promotion of root growth would help with the colonization of rice seedlings and its uptake of water to deal with drought.

## Discussion

Recent forecast have showed that global demand for food production will urgently need to double by 2050 [66]. This alarming prediction becomes more dire since the climate changes will probably cause a disruption of food production by extending drought events [10]. Together with the yield loss caused by plant disease, the challenges faced by plant pathologists and other agriculturalists seems really daunting. Recently, nanotechnology is starting to be regarded as a new weapon gradually in our arsenal against these increasing challenges in disease control [9]. However, the utilization of nanotechnology in plant disease management is still in its infancy and the specific molecular mechanisms remains largely unknown. Silica nanoparticles (SiO_2_ NPs) have recently been considered as one of the potential new safer agrochemicals whereas the systematically studies and specific mechanism remains unclear, especially in essential crops. Our results here found that in rice, one of the most important food crops, SiO_2_ NPs could enhance its resistance to the rice blast fungus *M. oryzae* through activating plant SA signaling. The inducing resistance of rice with SiO_2_ NPs foliar treatment was effective within a suitable concentration range under 120 mg/L, but has potential risk for aggravating the rice blast when the concentration reached 3000 mg/L high since too much nanoparticles intake. Not like the former studies, this kind of SiO_2_ NPs overdose caused disordered absorption in leaves had not been found before in rice [30, 67]. In contrast, root treatment showed better resistance through SAR response and better tolerance since the orderly and moderate absorption, indicating that root treatment is a safer and better application method of SiO_2_ NPs. Neither the effective concentration of foliar treatment (100 mg/L) nor the root treatment (3000 mg/L, 5 mg/g soil) exceed the reasonable application range according to previous studies [56–58], providing the possibility for the further use of SiO_2_ NPs. Moreover, root treatment through irrigating with SiO_2_ NPs promoted development of rice root and led to its capability upgrading of water absorption to cope with drought, which might play essential role in coping with the drought caused by climate changes.

Although silicon is not recognized as a pivotal element for general higher plants, it is beneficial to both plant production and resistance to biotic and abiotic stresses which used for fertilization in most cases [68, 69]. A supply of silicon to plants has been shown to reduce the intensities of several diseases in many economically important crops including rice [25]. Traditionally, silicon is uptake through plant roots as silicic acid depend on silicon transporters *Lsi1* and *Lsi2* in rice roots [24, 26, 70]. Combining our results and studies in other crops, it seemed that the intake of SiO_2_ NPs through root treatment probably more through the pores in the cell walls of the roots cells and then transport through inside root air spaces [60]. Furthermore, we revealed the mechanism of SiO_2_ NPs orderly intake by roots that blocked excessive nanoparticles outside the root epidermis.

The resistance of rice leaves to the *M. oryzae* through root treatment belongs to SA accumulation and SAR response. Salicylic acid (SA) is a key plant hormone that required for plant immunity including hypersensitive responses and SAR [71], and we found that the SiO_2_ NP-induced resistance to *M. oryzae* largely rely on SA signaling since not only the upregulation of SA-responsive genes, but also the block of induced resistance in SA defective mutants. Concurrently, the disordered intake under 3000 mg/l of foliar treatment also led to chaos of SA-related regulation which aggravated the disease. Although a recent study had found that SiO_2_ NPs can stimulate plant SAR between local leaves and systemic leaves in *A. thaliana* [30], we further extended the SAR range to that root treatment could enhance rice leaves resistance to the fungus and systematically established the response model of SAR response, SA content, as well as expressions of SA-regulated genes under exogenous SiO_2_ NPs treatment. Moreover, the several high concentrations of SiO_2_ NPs treatment did not lead to any toxic to the plant, showing its excellent application safety compared to traditional silicon [30].

## Conclusion

In summary, we revealed SiO_2_ NPs could stimulate plant immunity in order to enhance rice resistance to *M. oryzae* through SA-dependent pathway and confirmed root treatment as the effective and safe application method due to its reduction of phytotoxicity risk and promotion of the root development and water absorption in order to deal with adversity. Since amorphous SiO_2_ NPs have already are generally regarded as safe element that have been already used for dietary additives (E551) [72] in various of foodstuffs such as table salt, our findings here showed the good prospects of SiO_2_ NPs through root treatment, an effective and safe strategy for helping rice against biotic and abiotic stress which finally conducive to food production.

## Supplementary Information

**Additional file 1:** **Table S1.** Toxic test of SiO_2_NPs on fungi growth and appressorium formation. ^i^ Statistical analysis of the growth rate of *M. oryzae* with different treatment under 28 °C for 7 days and Duncan multiple range test was used for significance analysis. ^ii^ Percentage of conidial germination on artificial surface after 4 h. ^iii^ Percentage of appressorium formation on artificial surface after 24 h. Duncan’s new multiple range method p < 0.01. **Figure S1.** Fungal inhibition rate through foliar treatment under relative low concentrations of SiO_2_ NPs. Fungal growth inhibition rate was calculated by blast disease severity (evaluated by quantifying *M. oryzae* genomic 28S rDNA relative to rice genomic Rubq1 DNA) of (CK-treatment)/CK. Error bars represent SD. **Figure S2.** Toxicity test of SiO_2_ NPs on rice leaves. Different concentrations of SiO_2_ NPs were sprayed onto rice leaves for foliar treatment and the showed no significant toxicity after 7 days. CK stands for the control. **Figure S3.** TEM observation of non-treated control rice leaves. Leaves of the 2-week-old rice seedlings were sprayed with ultrapure water with spraying method treated for 1 day before observation. Ep stands for epidermis. The boxes with dashed and solid lines represent the magnification of the part. **Figure S4.** TEM observation of non-treated control rice roots. Roots of the 2-week-old rice seedlings were exposed to ultrapure water treated for 1 day before observation. Ep stands for epidermis, Ex stands for exodermis. The boxes with dashed and solid lines represent the magnification of the part. **Figure S5.** Detection of plant total silicon content under different treatments. Silicon concentration of each sample was detected at one day after foliar and root treatment with different concentrations of SiO_2_ NPs. Error bars represent SD and different capital letters represent significant differences (P < 0.01). **Figure S6.** Device for measuring drought resistance of rice. The device mainly contained a cylinder wall, a base part and seven rows of soil sample collection holes **A** and the 3D simulation rendering was made by SketchUp software **B**. **Figure S7.** Detection of soil water content. The water content was measured before the drought treatment in different soil layers and showed no significant differences between different layers. Error bars represent SD.

## Data Availability

All data generated or analyzed during this study are included in this published article.
