# Supplementary material for: Silica nanoparticles protect rice against biotic and abiotic stresses
Source: J Nanobiotechnology. 2022 Apr 22;20:197. doi: 10.1186/s12951-022-01420-x (PMC9034512; doi:10.1186/s12951-022-01420-x)
Supplement: Supplementary file 1 — Additional file 1: Table S1. Toxic test of SiO2NPs on fungi growth and appressorium formation. i Statistical analysis of the growth rate of M. oryzae with different treatment under 28 °C for 7 days and Duncan multiple range test was used for significance analysis. ii Percentage of conidial germination on artificial surface after 4 h. iii Percentage of appressorium formation on artificial surface after 24 h. Duncan’s new multiple range method p < 0.01. Figure S1. Fungal inhibition rate through foliar treatment under relative low concentrations of SiO2 NPs. Fungal growth inhibition rate was calculated by blast disease severity (evaluated by quantifying M. oryzae genomic 28S rDNA relative to rice genomic Rubq1 DNA) of (CK-treatment)/CK. Error bars represent SD. Figure S2. Toxicity test of SiO2 NPs on rice leaves. Different concentrations of SiO2 NPs were sprayed onto rice leaves for foliar treatment and the showed no significant toxicity after 7 days. CK stands for the control. Figure S3. TEM observation of non-treated control rice leaves. Leaves of the 2-week-old rice seedlings were sprayed with ultrapure water with spraying method treated for 1 day before observation. Ep stands for epidermis. The boxes with dashed and solid lines represent the magnification of the part. Figure S4. TEM observation of non-treated control rice roots. Roots of the 2-week-old rice seedlings were exposed to ultrapure water treated for 1 day before observation. Ep stands for epidermis, Ex stands for exodermis. The boxes with dashed and solid lines represent the magnification of the part. Figure S5. Detection of plant total silicon content under different treatments. Silicon concentration of each sample was detected at one day after foliar and root treatment with different concentrations of SiO2 NPs. Error bars represent SD and different capital letters represent significant differences (P < 0.01). Figure S6. Device for measuring drought resistance of rice. The device mainly contained a cyli [file 12951_2022_1420_MOESM1_ESM.pdf]

Treatment	Growth rate (cm)[i]	Germination rate (%)[ii]	Appressorium formation rate (%)[iii]
CK	5.1±1.2A	98.3±1.3A	97.5±1.5A
10 mg/L SiO_2 NPs	4.9±1.4A	98.1±1.7A	97.1±1.1A
100 mg/L SiO_2 NPs	5.2±1.4A	97.2±1.6A	98.6±1.5A
1000 mg/L SiO_2 NPs	4.9±1.6A	97.8±1.4A	96.8±1.6A
3000 mg/L SiO_2 NPs	5.0±1.2A	97.7±1.7A	97.7±1.3A

Table S1 Toxic test of SiO_2NPs on fungi growth and appressorium formation.

[i] Statistical analysis of the growth rate of *M. oryzae* with different treatment under 28°C for 7 days.

[ii] Percentage of conidial germination on artificial surface after 4 h.

[iii] Percentage of appressorium formation on artificial surface after 24 h.

Duncan's new multiple range method $p < 0.01$.

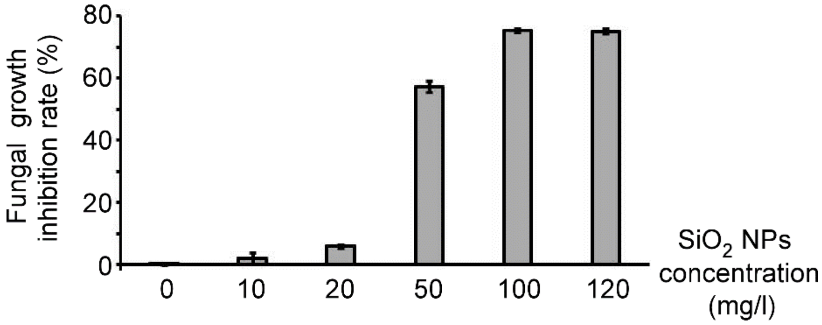

Figure S1. Fungal inhibition rate through foliar treatment under relative low concentrations of SiO_2 NPs. Fungal growth inhibition rate was calculated by blast disease severity (evaluated by quantifying *M. oryzae* genomic 28S rDNA relative to rice genomic Rubq1 DNA) of (CK-treatment)/CK. Error bars represent SD.

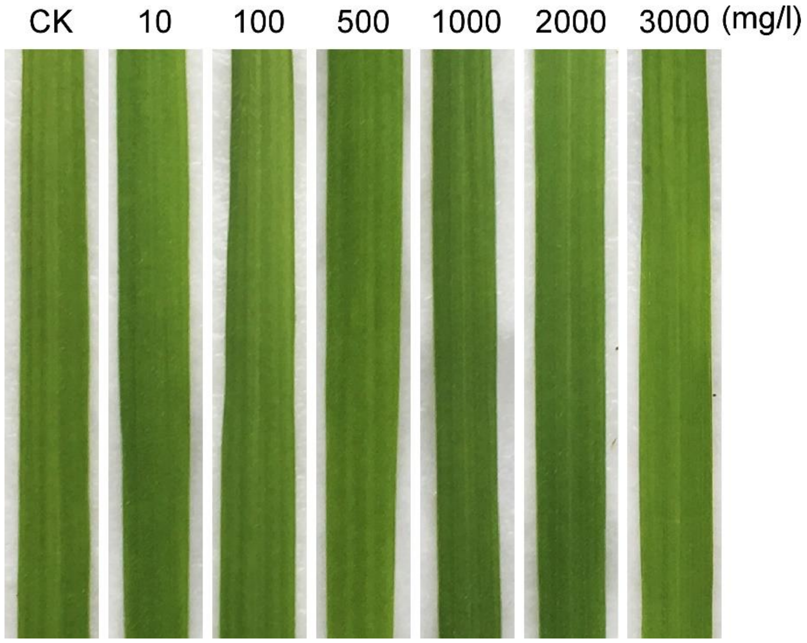

Figure S2. Toxicity test of SiO_2 NPs on rice leaves. Different concentrations of SiO_2 NPs were sprayed onto rice leaves for foliar treatment and the showed no significant toxicity after 7 days. CK stands for the control.

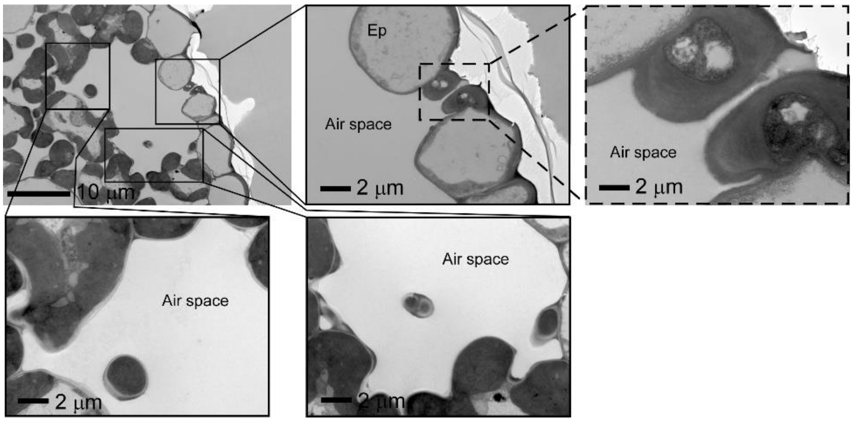

Figure S3. TEM observation of non-treated control rice leaves. Leaves of the 2-week-old rice seedlings were sprayed with ultrapure water with spraying method treated for 1 day before observation. Ep stands for epidermis. The boxes with dashed and solid lines represent the magnification of the part.

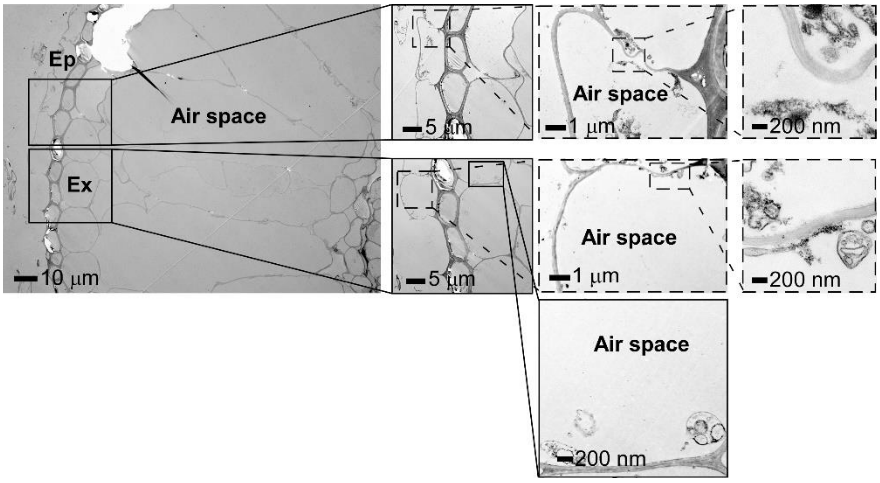

Figure S4. TEM observation of non-treated control rice roots. Roots of the 2-week-old rice seedlings were exposed to ultrapure water treated for 1 day before observation. Ep stands for epidermis, Ex stands for exodermis. The boxes with dashed and solid lines represent the magnification of the part.

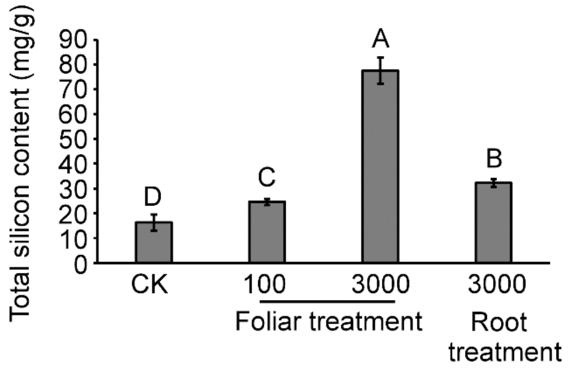

Figure S5. Detection of plant total silicon content under different treatments. Silicon concentration of each sample was detected at one day after foliar and root treatment with different concentrations of SiO_2 NPs. Error bars represent SD and different capital letters represent significant differences ($P < 0.01$).

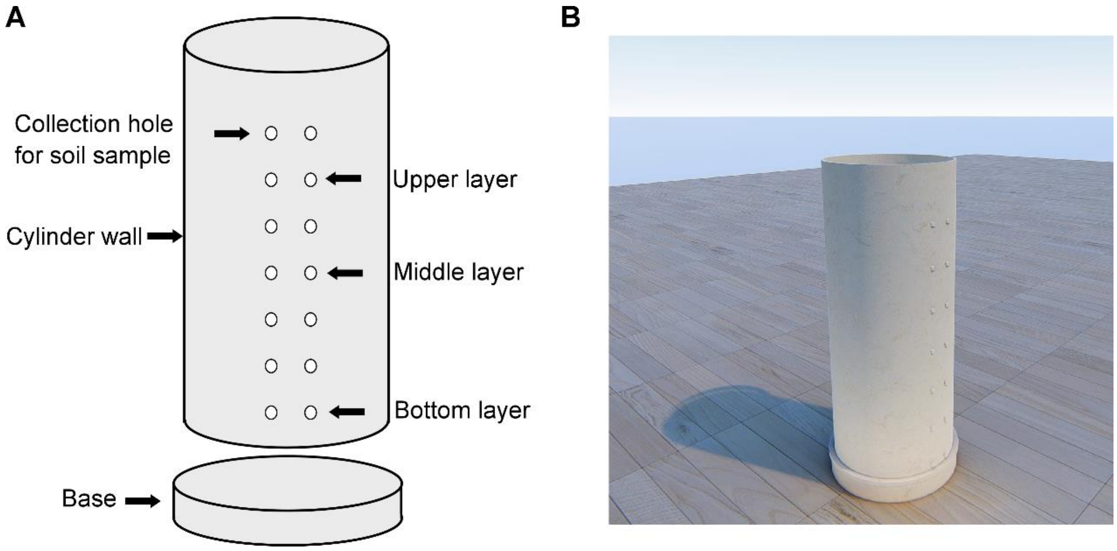

Figure S6. Device for measuring drought resistance of rice. The device mainly contained a cylinder wall, a base part and seven rows of soil sample collection holes **A** and the 3D simulation rendering was made by SketchUp software **B**.

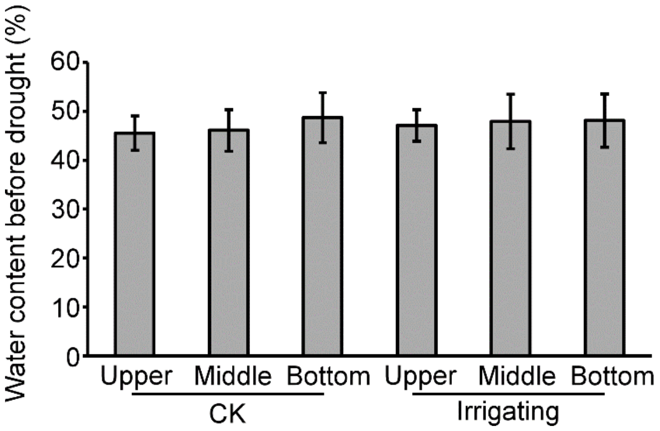

Figure S7. Detection of soil water content. The water content was measured before the drought treatment in different soil layers and showed no significant differences between different layers. Error bars represent SD.
